# A cationic tetrapyrrole inhibits toxic activities of the cellular prion protein

**DOI:** 10.1038/srep23180

**Published:** 2016-03-15

**Authors:** Tania Massignan, Sara Cimini, Claudia Stincardini, Milica Cerovic, Ilaria Vanni, Saioa R. Elezgarai, Jorge Moreno, Matteo Stravalaci, Alessandro Negro, Valeria Sangiovanni, Elena Restelli, Geraldina Riccardi, Marco Gobbi, Joaquín Castilla, Tiziana Borsello, Romolo Nonno, Emiliano Biasini

**Affiliations:** 1 Department of Molecular Biochemistry and Pharmacology, IRCCS-Istituto di Ricerche Farmacologiche Mario Negri, 20156 Milan, Italy; 2 Department of Neuroscience, IRCCS-Istituto di Ricerche Farmacologiche Mario Negri, 20156 Milan, Italy; 3 Department of Food Safety and Veterinary Health, Istituto Superiore di Sanitá, 00161 Rome, Italy; 4 CIC bioGUNE, Parque tecnológico de Bizkaia, Derio 48160, Bizkaia, Spain; 5 Department of Biomedical Sciences, University of Padova, 35121 Padova, Italy; 6 IKERBASQUE, Basque Foundation for Science, Bilbao 48013, Bizkaia, Spain; 7 Department of Pharmacological and Biomolecular Sciences, Milan University, 20133 Milan Italy; 8 Dulbecco Telethon Institute, Laboratory of Prions and Amyloids, Centre for Integrative Biology (CIBIO), University of Trento, 38123 Trento, Italy

## Abstract

Prion diseases are rare neurodegenerative conditions associated with the conformational conversion of the cellular prion protein (PrP^C^) into PrP^Sc^, a self-replicating isoform (prion) that accumulates in the central nervous system of affected individuals. The structure of PrP^Sc^ is poorly defined, and likely to be heterogeneous, as suggested by the existence of different prion strains. The latter represents a relevant problem for therapy in prion diseases, as some potent anti-prion compounds have shown strain-specificity. Designing therapeutics that target PrP^C^ may provide an opportunity to overcome these problems. PrP^C^ ligands may theoretically inhibit the replication of multiple prion strains, by acting on the common substrate of any prion replication reaction. Here, we characterized the properties of a cationic tetrapyrrole [Fe(III)-TMPyP], which was previously shown to bind PrP^C^, and inhibit the replication of a mouse prion strain. We report that the compound is active against multiple prion strains *in vitro* and in cells. Interestingly, we also find that Fe(III)-TMPyP inhibits several PrP^C^-related toxic activities, including the channel-forming ability of a PrP mutant, and the PrP^C^-dependent synaptotoxicity of amyloid-β (Aβ) oligomers, which are associated with Alzheimer’s Disease. These results demonstrate that molecules binding to PrP^C^ may produce a dual effect of blocking prion replication and inhibiting PrP^C^-mediated toxicity.

Prion diseases, which include Creutzfeldt-Jakob disease (CJD), fatal familial insomnia (FFI) and Gerstmann-Sträussler-Scheinker (GSS) syndrome, can manifest in a sporadic, inherited or transmissible fashion. These disorders are associated with the conformational conversion of PrP^C^, an endogenous cell-surface glycoprotein, into PrP^Sc^, a self-propagating, infectious protein (prion). PrP^Sc^ replicates by directly binding to PrP^C^, and causing its conformational rearrangement into new PrP^Sc^ molecules^1^. A great deal of evidence indicates that PrP^Sc^ may exist as an ensemble of conformers (referred to as prion strains), eliciting different neuropathological effects^2^. Prion strains represent a critical problem for treating prion diseases. In fact, several potent anti-prion compounds are strain-specific^3^,^4^,^5^. Moreover, acquisition of resistance to therapeutic treatments, reported in prion-infected cells and mice, has been attributed to the appearance of drug-resistant prion strains^6^,^7^. An additional confounding factor for drug discovery in prion diseases is related to the pathogenicity of PrP^Sc^. It is becoming increasingly evident that PrP^Sc^ is not neurotoxic per se, and instead requires functional PrP^C^ at the neuronal surface to deliver its detrimental effects^8^,^9^,^10^. Thus, PrP^C^ appears to play two crucial roles in prion diseases, by passively sustaining prion replication, and actively mediating PrP^Sc^ toxicity. Analogously, several studies have shown that PrP^C^ may act as a selective, high affinity and toxicity-transducing receptor for Aβ oligomers, which are thought to be responsible for the synaptotoxicity underlying the cognitive decline in Alzheimer’s disease^11^. An additional study reported that PrP^C^ may also mediate the cytotoxicity of other β-sheet-rich protein aggregates^12^. These data suggest that, in addition to PrP^Sc^, multiple disease-associated protein aggregates may use PrP^C^ to deliver their detrimental effects. This conclusion has therapeutic relevance. Compounds targeting PrP^C^, and blocking its transducing activity, may provide potential benefits for prion diseases, and possibly other neurodegenerative disorders^13^. Various chemical classes have been reported to bind PrP^C^. However, a careful evaluation of data reproducibility, as well as consistency between binding affinity and biological activity, restricted the number to a few^14^,^15^. Among these, an iron tetrapyrrole derivative [Fe(III)-TMPyP, Fe(III)-meso-tetra(N-methyl-4-pyridyl)porphine] was shown to interact with the C-terminal, structured domain of PrP^C^, and to inhibit prion replication *in vitro* and in cells^16^,^17^. The compound, or highly similar porphyrins, also significantly prolonged survival in prion-infected mice^18^,^19^,^20^. In this study, in addition to reproducing and extend PrP^C^-binding and anti-prion properties of Fe(III)-TMPyP, we report unexpected evidence regarding the activity of this compound in different cell-based assays for PrP^C^-related toxicity.

## Results

### Fe(III)-TMPyP binds to mouse, recombinant PrP^C^

**Figure 1:**
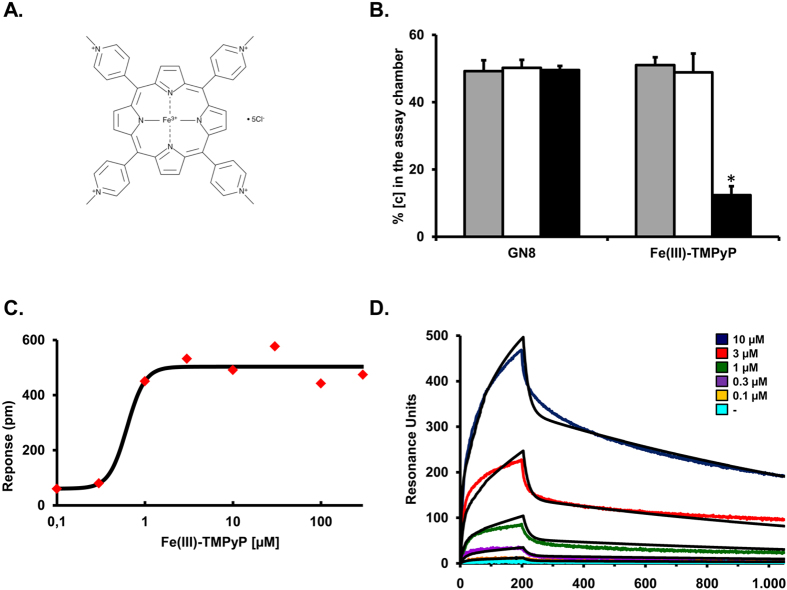
Fe(III)-TMPyP binds to PrP^C^. (**A**) Structure of Fe(III)-TMPyP; (**B**) Fe(III)-TMPyP but not GN8, binds to recombinant PrP^C^, as assayed by equilibrium dialysis. A 50 μM aliquot of the indicated compounds and recombinant proteins were placed in the assay and sample chambers, respectively. The chambers were separated by a 5,000 molecular weight cut-off membrane. Samples were left to equilibrate at room temperature for 1 day with gentle rocking. The concentration of each compound was quantified using UV-visible spectroscopy with buffer background subtracted. Statistics (by student *t*-test) was as follow: for GN8, Buffer vs BSA, *p* = 0.233; Buffer vs PrP^C^, *p* = 0.394; for Fe(III)-TMPyP, Buffer vs BSA, *p* = 0.129; Buffer vs PrP^C^, *p* = 3.9 × 10^−5^ (*). (**C**) Fe(III)-TMPyP binds to recombinant PrP^C^, as detected by DMR. Different concentrations of Fe(III)-TMPyP were added to label-free microplate well surfaces (EnSpire-LFB HS microplate, Perkin Elmer) on which full-length mouse recombinant PrP^C^ had previously been immobilized. Measurements were performed before (baseline) and after (final) adding the compound. The response (pm) was obtained subtracting the baseline output to the final output signals. The output signal for each well was obtained by subtracting the signal of the protein-coated reference area to the signal of uncoated area. The data (red dots) were fitted (black line) to a sigmoidal function using a 4 parameter logistic (4PL) nonlinear regression model; R^2^ = 0.96; *p* = 4.4 × 10^−3^. (**D**) SPR analyses of Fe(III)-TMPyP interaction with PrP^C^. Starting at time 0, the indicated concentrations of Fe(III)-TMPyP were injected for 200 sec over sensor chip surfaces (GL-H chip, Bio-Rad) on which 16.000 resonance units (RU) of full-length, mouse recombinant PrP^C^ had previously been captured by amine coupling. The chip was then washed with PBST buffer alone to monitor ligand dissociation. Sensorgrams show Fe(III)-TMPyP binding in RU. The data were obtained by subtracting the reference channels, and best fitted by the Langmuir equation, assuming a heterogeneous ligand on the surface. Kinetic constants were as follow: k_*a*_ = 5.95 × 10^2^ 1/Ms; k_*d*_ = 7.18 × 10^−4^ 1/s; K_d_ = 1.21 μM; R_max_ = 500.57 RU.

The cationic tetrapyrrole Fe(III)-TMPyP (Fig. 1A) was previously shown to bind human recombinant PrP^C^, and inhibit the replication of a mouse prion *in vitro* and in cells, by acting as a pharmacological chaperone for the native fold of the protein^17^. Here, we sought to confirm directly that Fe(III)-TMPyP is also able to bind full-length, mouse recombinant PrP^C^. First, we employed equilibrium dialysis, a technique originally used to detect binding of Fe(III)-TMPyP to human PrP^C^. The assay is based on the ability of a small molecule to equilibrate between two chambers, one filled with just buffer (assay chamber), and the other containing the target protein (sample chamber), separated by a membrane permeable only to the small molecule. As expected, Fe(III)-TMPyP (10 μM) equilibrated equally between the two chambers when the sample chamber contained no polypeptide, or BSA (10 μM). Conversely, when mouse recombinant PrP^C^ (10 μM) was added to the sample chamber, we observed a marked (>75%) decrease in the concentration of Fe(III)-TMPyP in the assay chamber, indicating that the compound interacted with PrP^C^ (Fig. 1B). No binding was observed for GN8, a small molecule originally claimed to bind PrP^C ^21, but that also failed to bind recombinant PrP^C^ in following studies^17^. Next, we employed dynamic mass redistribution (DMR), a label-free, biophysical technique previously employed to detect molecular interactions at the equilibrium (Fig. 1C)^22^,^23^. Recombinant, mouse PrP^C^ was immobilized on the surface of a 384-well, label-free microplate by amine-coupling chemistry. The plate was then incubated for 3 hr at room temperature to obtain optimal binding conditions, before adding different concentrations of Fe(III)-TMPyP (0.1–500 μM). Binding to PrP^C^ was monitored after an additional 30 min of incubation. We observed dose-dependent binding of Fe(III)-TMPyP to PrP^C^ in the concentration range of 0.1–1 μM, after which binding appeared to saturate. Experimental controls, used to normalize the signals, included empty surfaces (built in each microplate well) and buffer injections. Interestingly, the affinity constant obtained by DMR (Kd = 0.63 μM) was highly consistent with the inhibitory concentration at 50% (IC50) of Fe(III)-TMPyP and closely related compounds against prion replication in cell cultures (0.5–1 μM)^16^.

Finally, we employed SPR, another biophysical technique previously used to characterize association and dissociation constants in a kinetic manner (Fig. 1D). Recombinant PrP^C^ was immobilized on the surface of an SPR chip, as confirmed by binding of an anti-PrP antibody (not shown). We then monitored the association and dissociation of Fe(III)-TMPyP (0.1–10 μM) over the course of ~18 min. Non-specific interactions were accounted by subtracting signals from channels where only buffer was injected, or no polypeptides were immobilized. The sensorgrams were best fitted by a Langmuir binding model for a heterogeneous ligand, likely reflecting the presence of recombinant PrP^C^ molecules immobilized in different orientations onto the chip surface. These analyses indicated a dissociation constant for the interaction of Fe(III)-TMPyP to recombinant PrP^C^ of 1.21 μM. Once again, we failed to observe detectable binding (>100 μM) for GN8 (not shown). Collectively, these data confirmed that Fe(III)-TMPyP binds to recombinant mouse PrP^C^ in the low micromolar range. Of note, both DMR and SPR analyses revealed that Fe(III)-TMPyP binds also to BSA, used in some experiment to monitor non-specific interactions, at concentrations around 10 μM (not shown).

### Fe(III)-TMPyP inhibits the replication of different prion strains

**Figure 2:**
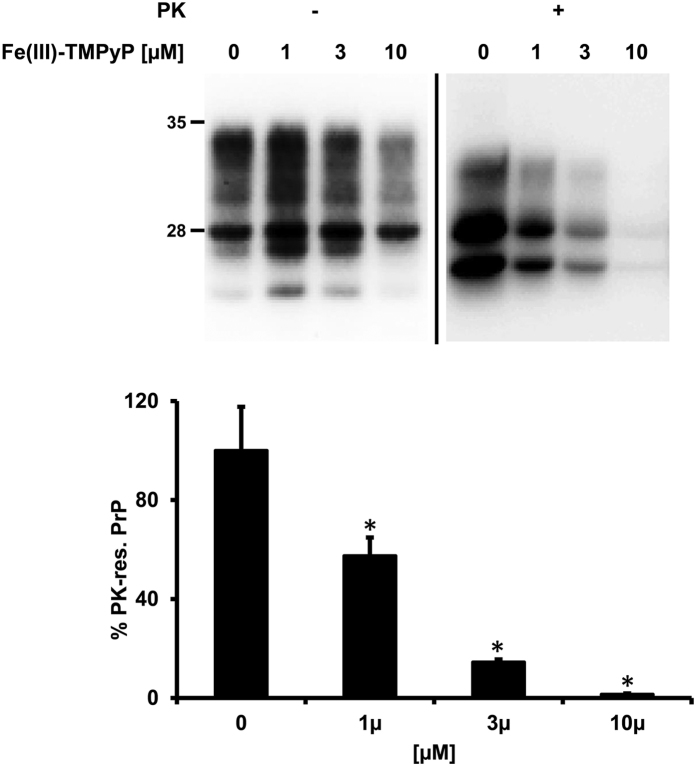
Fe(III)-TMPyP inhibits the replication of 22L prions in cell cultures. 22L-infected N2a cells were incubated with various concentrations (indicated) of Fe(III)-TMPyP for 72 h. The total amount of PrP^Sc^ was estimated in cell lysates by detecting the amount of protease K (PK)-resistant PrP by Western blot (upper panel), and quantifying signals by densitometric analysis (graph in the lower panel). Total PrP signal was revealed with anti-PrP^C^ antibody 6D11. Statistically-significant differences (*) between Fe(III)-TMPyP-treated and the untreated samples was estimated by student *t*-test: [1 μM], *p* = 0.034; [3 μM], *p* = 6.1 × 10^−6^; [10 μM], *p* = 3.4 × 10^−7^.

It was previously reported that Fe(III)-TMPyP inhibits the replication of the Rocky Mountain Laboratory (RML) prion strain in cells^16^,^17^. Since the compound has been proposed to act as a pharmacological chaperone, stabilizing the native conformation of PrP^C^, its inhibitory effect should theoretically be observed against multiple prion strains. In order to test this hypothesis, we first employed N2a cells, a sub-clone of murine neuroblastoma cells, infected with the 22L mouse prion strain. 22L-N2a cells were exposed for 72 h to different concentrations of Fe(III)-TMPyP (1–10 μM), or vehicle control, and the levels of proteinase K (PK)-resistant PrP^Sc^ were measured by Western blot. We confirmed that treatment with Fe(III)-TMPyP reduced 22L-prion loads in a dose-dependent fashion, with an IC_50_ of 1.61 ± 0.79 μM (Fig. 2). No cytotoxicity was observed at the active concentrations (not shown; lethal dose 50% was higher than 100 μM).

**Figure 3:**
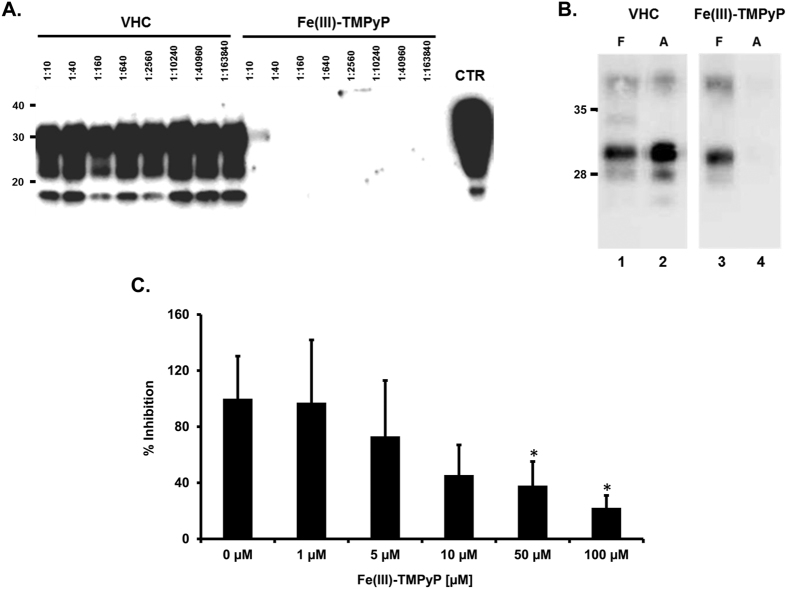
Fe(III)-TMPyP inhibits *in vitro* replication of vole-adapted scrapie. (**A**) Tg338 brain homogenates, seeded with indicated dilutions of Dawson isolate scrapie strain, were subjected to a single, 48 hr-long round of standard PMCA, upon incubation with Fe(III)-TMPyP (500 μM) or vehicle (VHC) control. Amplified samples were digested with PK and analyzed by Western blot using monoclonal antibody SAF83. Fe(III)-TMPyP showed an inhibitory activity toward prion amplification of at least 16,000 folds. CTR: Normal, unseeded and untreated brain homogenate. Molecular markers are KDa. (**B**) Brain homogenates from terminally ill voles infected with an Italian vole-adapted scrapie strain were diluted 1:10 (F, lanes 1 and 3) or 1:100 (A, lanes 2 and 4) in PMCA substrate in presence of vehicle (VHC, lanes 1–2) or 500 μM Fe(III)-TMPyP (lanes 3 and 4). Samples diluted 1:100 were subjected to a single PMCA round, while those diluted 1:10 were kept frozen and used to determine the amplification factor. Samples were PK-digested and analyzed by Western Blotting with antibody SAF84. (**C**) A similar experiment was carried out, in triplicate (n = 3), using different concentrations of Fe(III)-TMPyP (1–100 μM). The graph illustrates mean amplification factors (±standard error) obtained with increasing concentrations of Fe(III)-TMPyP or vehicle alone. Statistical differences (*) between Fe(III)-TMPyP and vehicle control were estimated by student *t*-test: [1 μM], *p* = 0,476; [5 μM], *p* = 0,273; [10 μM], *p* = 0,073; [50 μM], *p* = 0,047; [100 μM], *p* = 0,019.

To confirm that Fe(III)-TMPyP exerts its anti-prion effects in a strain-independent fashion, we employed the protein misfolding cyclic amplification (PMCA) reaction, a widely established *in vitro* assay for evaluating the propagation of prions in a test tube (Fig. 3)^24^. The methodology is based on a PrP^Sc^-triggered conversion of PrP^C^ by repetitive cycles of incubation and sonication. Brain lysates from transgenic (Tg) mice expressing ovine PrP (called Tg338) were used as a substrate for the amplification of scrapie prions, used as seeds (Dawson isolate). Different dilutions of scrapie (from 1:10 to 1:163, 840) were pre-incubated with Fe(III)-TMPyP (500 μM) or vehicle (control) and subjected to a single round of PMCA. The levels of PrP^Sc^ in each sample were then estimated by detecting PK-resistant PrP molecules by Western blot. In control samples, we observed robust PrP^Sc^ amplification at every dilution (Fig. 3A). Conversely, in presence of Fe(III)-TMPyP, PrP^Sc^ was barely detectable only at the lower dilution. We concluded that the compound exhibited a potent inhibitory activity toward the amplification of PrP^Sc^. To further substantiate this observation, we employed a PMCA assay specifically designed to rapidly test the inhibitory activity of small compounds. In this assay, 1:100 dilutions of bank vole-adapted prion strains are used as seeds for bank vole brain substrates, subjected to a single overnight PMCA round, in presence or absence of the test compound. By using this alternative PMCA protocol, we were able to generate a >10 fold amplification of PrP^Sc^ in 16 hours. At first, we determined the amplification factor of an Italian vole-adapted scrapie strain in presence of Fe(III)-TMPyP (500 μM) or vehicle control (Fig. 3B). In these conditions, Fe(III)-TMPyP showed strong inhibitory activity toward the amplification of vole-adapted PrP^Sc^ (amplification factors dropped from 14.43 ± 3.47 to 1.46 ± 0.83). Of note, exposure to Fe(III)-TMPyP did not alter the detection of PK-resistant PrP molecules by Western blot, as evidenced by the similar levels of PrP^Sc^ detected in the frozen (F) controls (Fig. 3B, lanes 1 and 3). We tested the molecule also against two additional bank vole-adapted scrapie strains, distinct from the Italian strain [namely, a UK strain, derived from the sheep isolate SCR1^25^, and vole-adapted CH1641^26^]. Fe(III)-TMPyP (500 μM) showed a comparably potent inhibitory effect against the three different strains (not shown). Finally, we performed dose-response experiments to estimate the IC_50_ of Fe(III)-TMPyP against the Italian vole-adapted scrapie strain (Fig. 3C). We found that the compound was already active at low micromolar concentrations (IC_50_ = 6.21 + /−1.9 μM). However, statistical significance was observed only at higher concentrations (50–100 μM), possibly due to the high variability of the assay. Taken together, these data strongly supported the notion that Fe(III)-TMPyP exerts its anti-prion activity in a strain-independent fashion.

### Fe(III)-TMPyP inhibits the cytotoxic effects of a mutant PrP

**Figure 4:**
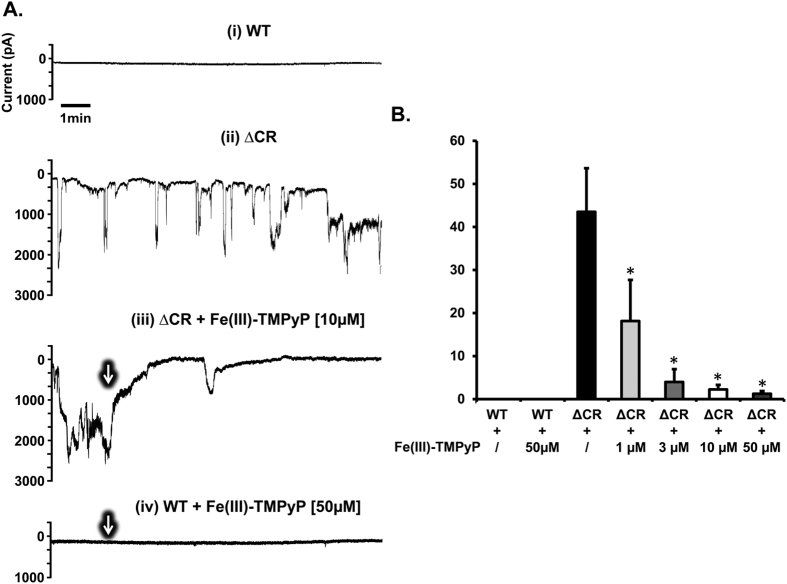
Fe(III)-TMPyP inhibits ionic currents induced by ΔCR PrP. (**A**) Whole‐cell patch clamp recording from HEK293 cells expressing either WT or ΔCR PrP, at a holding potential of −80 mV. Fe(III)-TMPyP at the reported concentrations was added to the dish at the time indicated by the arrows. (**B)** Inward currents recorded from WT or ΔCR PrP HEK293 cells were plotted as the percentage of total time the cells exhibited currents ≥450 pA (mean ± S.E.M., n ≥ 5 cells), at a holding potential of −80 mV. Statistically-significant differences (*) between Fe(III)-TMPyP-treated and the untreated ΔCR cells were estimated by student *t*-test: [1], *p* = 0.036; [3], *p* = 0.0016; [10], *p* = 0.0014; [50], *p* = 0.005.

We have previously reported that PrP^C^ molecules carrying deletions or point mutations in the conserved central region (CR) are associated with a spontaneous ion channel activity that critically depends on the conserved polybasic sequence at the extreme N-terminus^27^,^28^. These currents can be easily recorded by patch clamping techniques. Since Fe(III)-TMPyP binds directly to PrP^C^, we tested whether this compound could alter the ion channel activity of ∆CR PrP (deleted for residues 105–125), the most active among the channel-forming mutants. As expected, HEK293 cells expressing ΔCR PrP displayed large, spontaneous inward currents that were absent in cells expressing wild-type (WT) PrP (Fig. 4). Importantly, the currents were dose-dependently silenced by treatment with Fe(III)-TMPyP (IC_50_ = 1.7 μM). The compound showed no effects at the highest concentration (50 μM) in WT cells.

**Figure 5:**
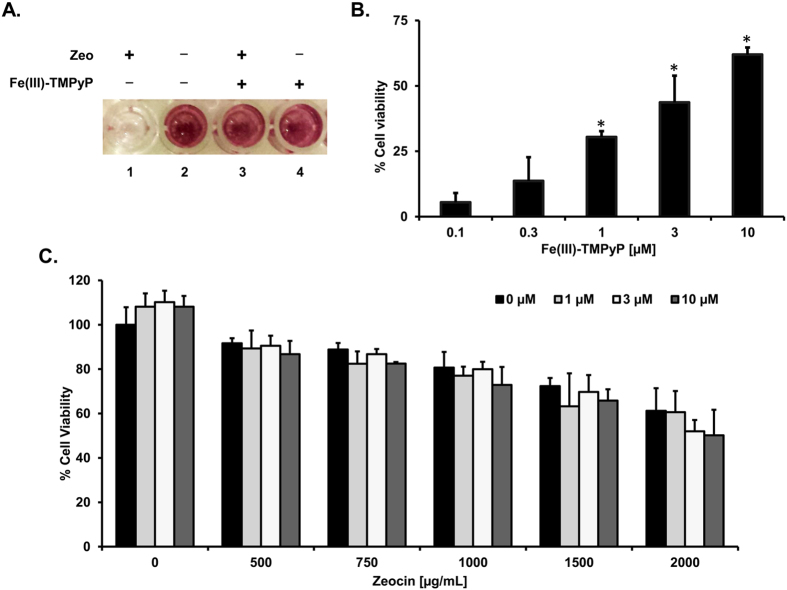
Fe(III)-TMPyP inhibits the drug-hypersensitizing effect of ΔCR PrP. (**A)** The DBCA was used to evaluate the anti-ΔCR PrP effects of Fe(III)-TMPyP. Stably transfected WT or ΔCR HEK293 cells carrying the hygromycin B resistance cassette were plated in 24-well plates and incubated in medium containing 500 μg/mL of Zeocin, for 48 h at 37 °C. The picture shows an example of treated vs untreated wells after MTT assay. (**B)** The bar graph shows a quantification of the dose-dependent, rescuing effect of Fe(III)-TMPyP. Average values were obtained from a minimum of 4 independent experiments (n = 4), and expressed as a percentage of cell viability in untreated cells. Statistically-significant differences (*) between Fe(III)-TMPyP-treated and untreated cells was estimated by student *t*-test: [0.1], *p* = 0.1582; [0.3], *p* = 0.0659; [1], *p* = 1.67 × 10^−4^; [3], *p* = 2.19 × 10^−4^; [10], *p* = 3.37 × 10^−6^. (**C**) Fe(III)-TMPyP did not rescue the toxicity of Zeocin in WT PrP-expressing cells. The DBCA was adapted to test the toxicity of Zeocin in HEK293 cells stably expressing WT PrP, and evaluate the potential rescuing effect of Fe(III)-TMPyP. Cells were plated in 24-well plates and incubated in medium containing different concentrations (0–2.000 μg/mL) of Zeocin, for 72 h at 37 °C, in presence (1–10 μM) or absence of Fe(III)-TMPyP. Average values were obtained from a minimum of 3 independent experiments (n = 3), and expressed as percentage of cell viability of Zeocin-untreated cells.

**Figure 6:**
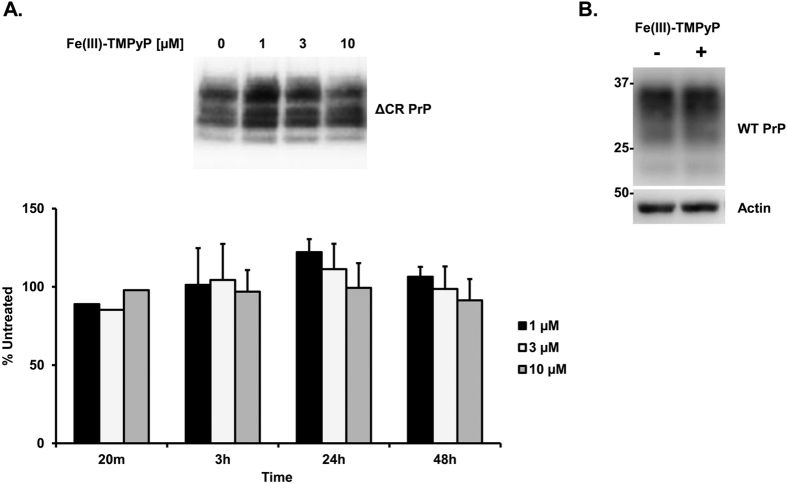
Treatment with Fe(III)-TMPyP does not alter PrP^C^ expression. (**A**) HEK293 cells expressing ΔCR PrP were treated with Fe(III)-TMPyP at different concentrations, and for the indicated time points. Total PrP levels were evaluated in whole-cell lysates by western blot, using anti-PrP antibody 6D11. The picture in the upper panel illustrates a Western blot related to the 3 h timepoint. Graph in the bottom panel shows the quantification of PrP levels, obtained by densitometric analysis of the Western blots, normalizing each value on the corresponding Ponceau S-stained lane. Bars represent the mean of two (20 min) to four (±SEM) independent experiments, expressed as percentage of the levels in untreated cells. (**B)** Hippocampal neurons from C57BL/6 mice were exposed to 10 μM Fe(III)-TMPyP (+) or the vehicle (−) for 24 h. Cells were lysed and analyzed by Western blot with anti-PrP (6D11) or anti-actin antibodies.

As a possible consequence of its channel-forming activity, expression of ∆CR PrP has been shown to hypersensitize various cultured cell lines to the toxicity of cationic antibiotics^29^. These include Zeocin, a member of the bleomycin/phleomycin family, and aminoglycoside G418. This peculiar property was previously exploited to develop a drug-dependent cellular assay (DBCA) for studying the toxicity of ∆CR PrP^30^. Here, we used the DBCA to confirm the inhibitory effect of Fe(III)-TMPyP. As expected, ΔCR-expressing HEK293 cells exposed to Zeocin (500 μg/mL; Fig. 5A) or G418 (500 μg/mL; not shown) for 72 h showed strongly reduced viability (<30%), as assayed by MTT. Consistent with patch-clamp recordings, co-treatment with Fe(III)-TMPyP (0.1–10 μM) produced a robust, dose-dependent rescue of antibiotic-induced cell death (IC_50_ = 3.26 ± 2.12 μM; Fig. 5B). The compound failed to rescue the non-specific cell death induced by high concentrations of Zeocin (up to 2 mg/mL) in WT cells (Fig. 5C). Fe(III)-TMPyP was also not toxic to control cells (up to 50 μM) and did not significantly alter ΔCR PrP expression (Fig. 6A) or cell surface localization (not shown). These results indicated that Fe(III)-TMPyP acts a specific inhibitor of ∆CR PrP activity.

### Fe(III)-TMPyP blocks the synaptotoxicity of Aβ oligomers

**Figure 7:**
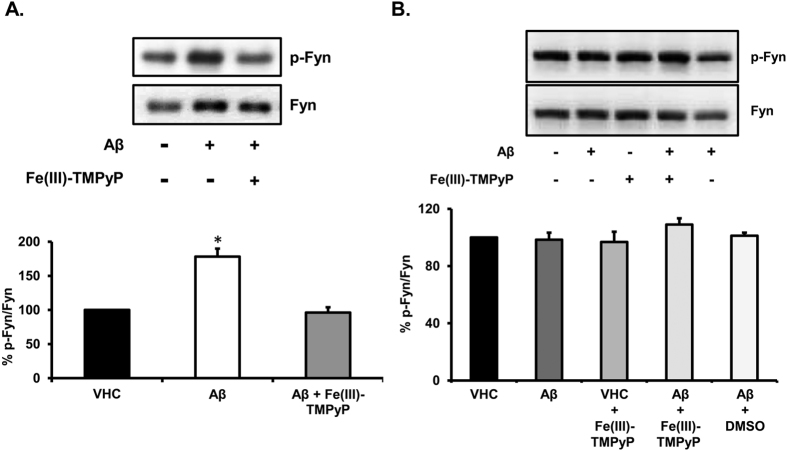
Fe(III)-TMPyP inhibits Aβ oligomer-dependent activation of Fyn. (**A**) Treatment with Aβ oligomers induced a rapid phosphorylation of the Fyn kinase, which was fully prevented by pre-incubation with Fe(III)-TMPyP. Primary hippocampal neurons were pre-treated for 20 minutes with Fe(III)-TMPyP (10 μM) or vehicle control (volume equivalent) prior to incubation with Aβ oligomers (3 μM, monomer equivalent concentration) for 20 minutes. Triton-insoluble fractions were analyzed by immunoblot with antibodies against phospho-SFK (Tyr 416) or total Fyn. Actin was used as loading control. The picture shows an example of a Western blot for p-Fyn and Fyn. The graph reports the quantification of 5 independent experiments (n = 5). Values are expressed as percentage of vehicle (VHC)-treated cells, normalized on the intensity of the corresponding Actin bands. *Statistical significance was estimated by one-way ANOVA, Tukey post-hoc test (p = 3.77 × 10^−4^). (**B)** Neither Aβ, nor Fe(III)-TMPyP altered the phosphorylation of Fyn in PrP-null neurons. Primary hippocampal neurons derived from PrP KO mice were pre-treated for 20 minutes with Fe(III)-TMPyP (10 μM) or DMSO (volume equivalent) prior to incubation with Aβ oligomers (3 μM, monomer equivalent concentration) or vehicle (VHC, volume equivalent) for 20 minutes. Triton-insoluble fractions were analyzed by immunoblot with antibodies against phospho-SFK (Tyr 416) or total Fyn. Actin was used as loading control. The upper panel shows an example of a Western blot for p-Fyn and total Fyn. The bar graph reports the quantification of four independent experiments (n = 4). Values are expressed as percentage of VHC-treated cells, normalized on the intensity of the corresponding actin bands.

**Figure 8:**
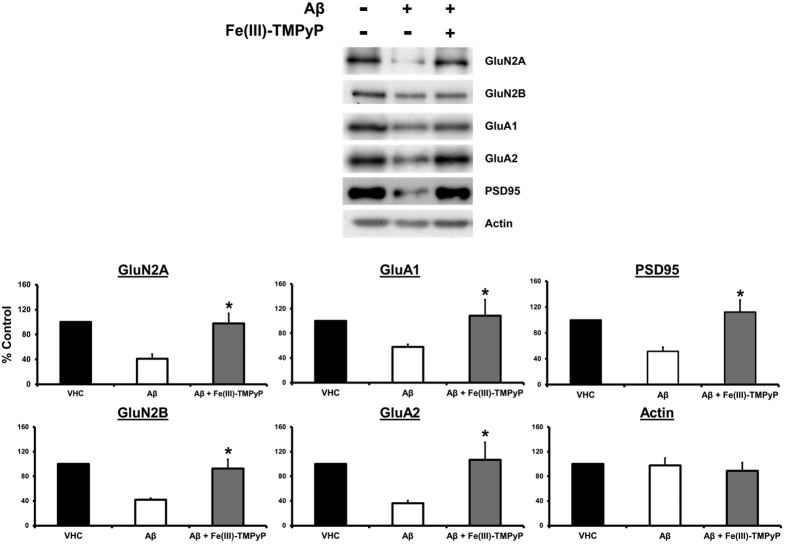
Fe(III)-TMPyP rescues the synaptotoxic effects of Aβ oligomers. Aβ oligomers induced a loss of post-synaptic markers, which was significantly attenuated by pre-incubation with Fe(III)-TMPyP. Primary hippocampal neurons were pre-treated for 20 min with or without Fe(III)-TMPyP (10 μM), and exposed for 3 h to Aβ oligomers (3 μM) or DMSO vehicle (VHC). Post-synaptic proteins from triton-insoluble fractions were then measured by Western blotting. The picture shows a representative Western blot for each synaptic marker. Quantitation of multiple experiments is shown in the graph. Actin levels were not significantly affected by Aβ oligomer treatment. *Statistical significance. P values, calculated by one-way ANOVA, Tukey post-hoc test, were as follow: GluN2A, p = 0.013542; GluN2B, p = 0.010834; GluA1, p = 0.002597; GluA2, p = 0.015558; PSD95, p = 0.036794.

The interaction between PrP^C^ and Aβ oligomers has been shown to trigger a rapid, toxic signaling pathway involving the transient activation of the tyrosine kinase Fyn, and ultimately producing a dysregulation of NMDA receptors, excitotoxicity, and dendritic spine loss^11^,^31^,^32^. Therefore, we tested whether Fe(III)-TMPyP could also inhibit the toxicity of Aβ oligomers mediated by PrP^C^. In a first set of experiments, we exposed primary cultures of postnatal mouse hippocampal neurons to Aβ oligomers (3 μM) for 20 minutes. Consistent with previous studies^32^, we found that the oligomers induced a quick, transient and robust (~2 fold) increase of phosphorylated Fyn (Fig. 7A). The phospho-SFK antibody used here detects pY416 in several SFKs, but previous studies demonstrated that PrP^C^-dependent activation of kinases is specific for Fyn^32^. Pre-incubation for 20 minutes with Fe(III)-TMPyP (10 μM) completely prevented Aβ effects, maintaining Fyn phosphorylation to normal levels (Fig. 7A). As observed in cultured cells, the compound showed no effect on PrP^C^ expression (Fig. 6B). Consistent with previous reports^32^, no significant increase in Fyn phosphorylation was detected in hippocampal neurons derived from PrP knockout (KO) mice, upon treatment with Aβ oligomers and/or Fe(III)-TMPyP (Fig. 7B). Next, we tested the ability of Fe(III)-TMPyP to block Aβ oligomer-dependent synaptotoxicity (Fig. 8). Primary hippocampal neurons were incubated for 3 hours with Aβ oligomers (3 μM). Consistent with previous reports^33^, we observed a decrease of several post-synaptic markers, as evaluated by Western blot of the Triton-insoluble fractions. Post-synaptic markers affected by Aβ oligomer treatment included subunits of the glutamate receptors NMDA (GluN2A, GluN2B, decreased to 40.9 ± 7.5 and 42.1 ± 2.3%, respectively) and AMPA (GluA1 and GluA2, decreased to 57.8 ± 4.3 and 36.1 ± 4.9%), as well as the post-synaptic density protein 95 (PSD-95; reduced to 51.3 ± 6.3%). In absence of Aβ oligomers, the levels of these proteins were not significantly changed upon treatment with Fe(III)-TMPyP (not shown). However, pre-treatment with Fe(III)-TMPyP for 20 minutes prior to incubation with Aβ oligomers significantly rescued the levels of all the post-synaptic markers (GluN2A and GluN2B levels were increased to 97,7 ± 16,3 and 92,6 ± 15%; GluA1 to 108.5 ± 25.9 GluA2 both to 106.7 ± 28.4%; and PSD95 to 112.2 ± 18.6%). The level of a control protein (actin) was not affected by either Aβ oligomers or Fe(III)-TMPyP. Collectively, these data demonstrate that Fe(III)-TMPyP inhibits the synaptotoxic effects of Aβ oligomers mediated by PrP^C^.

## Discussion

Mounting evidence suggests that PrP^Sc^ may be an inconvenient pharmacological target^34^. Indeed, several molecules identified as potent anti-prion agents in cells, have proven to be strain-dependent, showing therapeutic efficacy *in vivo* only against specific prion strains^5^,^35^. To further complicate the matter, poorly defined misfolded PrP intermediates, rather than fully aggregated PrP^Sc^ isoforms, are emerging as potential pathological species in prion diseases^36^,^37^. We and others have been interested to explore an experimental strategy that may overcome these problems by directly targeting PrP^C^ 13,38. Small molecules binding to PrP^C^ with sufficiently high affinity could theoretically stabilize its tertiary structure enough to disfavor the generation of any misfolded state or pathogenic PrP conformer. Moreover, since they target the native precursors of prion-induced misfolding reactions, rather than the aggregated end-products, PrP^C^-directed compounds should theoretically be effective independently of the prion strain phenomenon. A similar strategy applied to transthyretin, a protein associated with familial amyloid polyneuropathy, recently led to the development of Tafamidis, an approved drug for these disorders^39^,^40^. Multiple chemical classes have been reported in the literature to bind PrP^C^. These include tricyclic phenothiazines, 2,4-diarylthiazoles, quinacrine, a compound named GN8, pyridine-3,5-dicarbonitriles, tetrapyrroles, and few others^15^,^41^. Some of these, such as quinacrine, have been shown to bind PrP^C^ in a non-specific fashion^42^. Others, like GN8, have not been confirmed to be PrP^C^ ligands in following publications (we also failed to detect binding of GN8 to mouse PrP^C^ in this study)^17^. Additional molecules were shown to bind PrP^C^ at biologically-irrelevant concentrations. For example, binding to PrP^C^ for promazine or chlorpromazine was observed by using millimolar concentrations of compounds^43^. However, these molecules are effective against prion replication in cell cultures at low micromolar concentrations, making it difficult to reconcile their biological activity with their putative, PrP^C^-directed mode of action^44^,^45^. In this study, we sought to characterized the biological properties of a synthetic, iron-complexed porphyrin named Fe(III)-TMPyP. This compound was reported to interact with a relatively large region in the C-terminal, structured domain of PrP^C^, in correspondence to a pocket near the C-terminus of helix-3^17^. Fe(III)-TMPyP was shown to act as a pharmacological chaperone for PrP^C^, lowering the ground state of its native conformation, and inhibiting prion-induced misfolding *in vitro* and in cell cultures. Importantly, binding affinity for PrP^C^ and active concentrations against prion propagation were comparable (both in the low micromolar range). We confirmed binding of Fe(III)-TMPyP to recombinant PrP^C^ by three different techniques. These included equilibrium dialysis, which was the primary screen that led to the original identification of Fe(III)-TMPyP binding to PrP^C^, and two complementary biophysical techniques (DMR and SPR). The latter allowed us to estimate the binding affinity of Fe(III)-TMPyP for PrP^C^ in the low micromolar concentration range, consistent with previously reported affinity constants^17^. We also detected an interaction between Fe(III)-TMPyP and BSA when the compound was tested by DMR and SPR, at concentrations slightly higher than those active in biological assays. Curiously, this was not observed by equilibrium dialysis. The possible, non-specific interaction with such an abundant plasma protein, together with the unlikelihood that the compound may cross the blood brain barrier, could negatively affect the therapeutic potential of Fe(III)-TMPyP upon systemic administration. Therefore, extensive pharmacokinetic profiling, coupled to chemical optimization efforts, will be required to design effective drug-like derivatives of Fe(III)-TMPyP.

We also tested the hypothesis that Fe(III)-TMPyP inhibits prion propagation in a strain-independent manner. The compound was challenged against the 22L prion strain in neuroblastoma cells, and four different sheep prion strains by using two alternative PMCA protocols. We found comparable IC_50_ values for the activity of Fe(III)-TMPyP in cells and PMCA (1.61 μM and 6.21 μM, respectively). However, the inhibitory effect of the compound was much less reliable in the PMCA assay (statistically significant effects were detected only above 50 μM). Reasons for such discrepancy could be related to the stability of the compound in brain lysates under heavy sonication conditions, or simply to the higher variability of the PMCA assays. In any case, these data confirm the ability of the compound to bind PrP^C^ at biologically-relevant concentrations, and exert its inhibitory activity against multiple prion strains.

Based on the observation that Fe(III)-TMPyP is a PrP^C^ ligand, we were prompted to test the possibility that this compound may exert specific effects on PrP^C^ activity. Despite two decades of intense investigation, the physiological function of PrP^C^ is still uncertain^10^,^46^. However, several direct or indirect effects of PrP^C^ expression have been described in cells and mice. One of these is the ability of WT PrP^C^ to rescue the neurodegenerative phenotype caused by particular PrP mutants^47^. In an attempt to investigate the role of PrP^C^ in synaptic function, and how this activity could be connected to prion neurotoxicity, we and others have studied mutant forms of PrP carrying deletions in the highly conserved central region of the protein^27^,^48^,^49^. One of these mutants, referred to as ΔCR PrP (Δ105–125), has been shown to be soluble, protease-sensitive, and to have the same cellular localization pattern of WT PrP^C ^50. However, in contrast to PrP^C^, expression of ΔCR PrP in transgenic mice causes cerebellar degeneration and neonatal death^27^. The neurotoxic mechanism activated by ΔCR PrP may relate to its ability to induce ionic currents at the plasma membrane. These currents are the most likely cause of the hypersensitivity to cationic antibiotics observed in a variety of ΔCR PrP-expressing cells^29^. *In vivo*, ΔCR PrP currents alter the physiological homeostasis of glutamatergic synapses, causing the activation of an excitotoxic cascade, and early degeneration of the granule neurons in the cerebellum^51^. Importantly, the entire spectrum of detrimental effects caused by ΔCR PrP can be dose-dependently suppressed by the co-expression of WT PrP^C^, suggesting that the mutant and WT proteins could be interacting at a functional level^10^. ΔCR PrP has another intriguing property that suggests its relevance for PrP^C^ biology. Its activity has been shown to be dependent on two stretches of poly-basic residues in the N-terminal, flexible tail of the protein (23–28 and 95–105)^28^. Strikingly, these two sites are also directly involved in the interaction between PrP^C^ and Aβ oligomers, and at least two additional protein aggregates^12^,^52^. Together, these data suggest the intriguing possibility that the aberrant activity of ΔCR PrP, and the neurotoxic signaling activated by Aβ oligomers, could be connected to the physiological function of PrP^C^ 10,14. In this study, we show that Fe(III)-TMPyP blocks ΔCR PrP activity in a dose-dependent fashion in two different cellular assays. These included the suppression of ΔCR PrP-induced currents, as measured by whole-cell patch clamp, and the inhibition of the hypersensitivity to cationic antibiotics conferred by the mutant, as evaluated by DBCA. Moreover, we found that pre-incubation with Fe(III)-TMPyP protects primary hippocampal neurons to the Fyn-mediated, PrP^C^-dependent synaptotoxicity of Aβ oligomers. These protective effects of Fe(III)-TMPyP are somehow unexpected. In fact, the compound has been shown to bind a pocket located in the C-terminal domain of PrP^C^ 17. This region is far from the CR deletion, and opposite from the structural determinants of ΔCR PrP activity and Aβ binding sites, which are both located in the unstructured N-terminus. How does binding of Fe(III)-TMPyP in the C-terminus block the toxic activities of the PrP^C^ flexible N-terminus? One possibility is suggested by several recent studies indicating that the C-terminal and N-terminal domains of PrP^C^ may be functionally coupled, or even directly interact with each other^53^,^54^,^55^,^56^. Therefore, Fe(III)-TMPyP could be acting as an allosteric inhibitor of PrP^C^, blocking the toxic activities elicited by the N-terminal tail. This possibility opens up intriguing opportunities to employ Fe(III)-TMPyP not only as a therapeutic agent, but also as an experimental tool to study the function of PrP^C^.

In summary, we have employed multiple biochemical, biophysical and cell-based techniques to demonstrate that Fe(III)-TMPyP binds to PrP^C^, and acts as a strain-independent inhibitor of prion replication. Importantly, we also show that the compound blocks the aberrant channel-forming ability of mutant ΔCR PrP, and prevents the synaptotoxic effects delivered by Aβ oligomers. These data have important therapeutic implications for prion diseases, and possibly other neurodegenerative conditions. Drug-like, brain-penetrant derivatives of Fe(III)-TMPyP may block prion replication and inhibit PrP^C^-mediated toxicity. Moreover, these compounds could reveal important insights into the physiological activity of PrP^C^, and its functional connection to neurodegenerative pathways.

## Methods

### Ethics Statement

All experiments involving animals were carried out in accordance with European guidelines (Directive 2010/63/EU), and were approved by the animal experimentation ethics committee at CIC bioGUNE [in agreement with Article 28, sections a), b), c) and d) of the “Real Decreto 214/1997 de 30 de Julio”]. The experiments were also carried out in accordance with the guidelines established by the Italian legislation (L.D. no. 26/2014), reviewed by the Animal Welfare Committee of the University of Milan, and approved by the Italian Ministry of Health.

### Equilibrium dialysis

Compounds were diluted from a 50 mM stock into 1 mM sodium acetate buffer (pH 5). One hundred microliters of 50 μM Fe(III)-TMPyP, or GN8, were placed in the assay chamber of a 96-Well Equilibrium DIALYZER plate (Harvard Apparatus, Holliston, MA), with a 5,000 molecular weight cut-off membrane. One hundred microliters of buffer, 50 μM recombinant PrP^C^ or bovine serum albumin (BSA) were placed in the sample chamber. Recombinant PrP^C^ (residues 23–230) was expressed and purified as described previously^57^. Samples were left to equilibrate at room temperature for 24 h with slow rocking. The concentration of each compound from the assay chamber was quantified using a UV-visible spectrometer (Biotek, Winooski, VT), with the appropriate buffer or protein background subtraction.

### Dynamic Mass Redistribution (DMR)

The EnSight Multimode Plate Reader (Perkin Elmer, Waltham, MA) was used to carry out DMR analyses. Immobilization of full-length (residues 23–230), mouse recombinant PrP^C^ (15 μL/well of a 2.5 μM PrP^C^ solution in 10 mM sodium acetate buffer, pH 5) on label-free microplates (EnSpire-LFB high sensitivity microplates, Perkin Elmer) was obtained by amine-coupling chemistry. The interaction between Fe(III)-TMPyP, diluted to different concentrations in assay buffer (10 mM PO4, pH 7.5, 2.4 mM KCl, 138 mM NaCl, 0.05% Tween-20) and PrP^C^, was monitored after a 30 min incubation at room temperature. All the steps were executed by employing a Zephyr Compact Liquid Handling Workstation (Perkin Elmer). The Kaleido software (Perkin Elmer) was used to acquire and process the data.

### Surface plasmon resonance (SPR)

Binding studies were performed using the ProteOn XPR36 Protein Interaction Array system (Bio-Rad, Hercules, CA). The binding of Fe(III)-TMPyP was monitored by immobilizing ~16,000 Resonance Units (RU) of recombinant PrP^C^ on the surface of a sensor chip (GL-H chip, Bio-Rad) by amine-coupling chemistry. Compounds were perfused over the chip for 150 sec to allow association, followed by a buffer (10 mM PO4, pH 7.5, 2.4 mM KCl, 138 mM NaCl, 0.05% Tween-20) wash to monitor dissociation. The resulting sensorgrams (time course of resonance unit signal) were fitted using the ProteOn analysis software (Bio-Rad) to obtain the corresponding association and dissociation rate constants (k_on_ and k_off_, respectively), and the equilibrium dissociation constant (K_d_).

### Western blots

Samples were diluted 1:1 in 2X Laemli sample buffer (2% SDS, 10% glycerol, 100 mM Tris-HCl pH 6.8, 0.002% bromophenol blue, 100 mM DTT), heated at 95 °C for 10 min, then analyzed by SDS-PAGE. Proteins were electrophoretically transferred to polyvinylidene fluoride (PVDF) membranes, which were then blocked for 20 min in 5% (w/v) non-fat dry milk in Tris-buffered saline containing 0.05% Tween-20. After incubation with appropriate primary and secondary antibodies, signals were revealed using enhanced chemiluminescence (Luminata, BioRad), and visualized by a Bio-Rad XRS Chemidoc image scanner (Bio-Rad).

### PMCA

Two different versions of the PMCA reactions were used in this study. The first one was performed as described previously^24^, with minor modifications. Briefly, Tg338 brains used for substrate were perfused using PBS/5 mM EDTA, and frozen immediately. A 50 μl aliquot of 10% Tg338 brain homogenate, seeded with different dilutions of scrapie (Dawson isolate) were loaded onto 0.2-ml PCR tubes. Samples were incubated with 0.5 μl of PBS (not shown), DMSO or Fe(III)-TMPyP (at final concentration of 500 μM) and placed at 37–38 °C into a sonicating water bath without shaking. Tubes were positioned on an adaptor placed on the plate holder of the sonicator (model S-700MPX, QSonica, Newtown, CT, USA), and subjected to cycles of incubation (30 min) followed by a 20 s pulse of 150–220 watts sonication (at 70–90% of amplitude) for 48 hr. The detailed protocol for PMCA, including reagents, solutions and troubleshooting, has been published elsewhere^58^.

The second PMCA assay was performed as described previously^59^, with minor modifications. Briefly, PMCA substrates were prepared using 2–3 month-old bank voles homozygous for methionine at codon 109 (Bv109M). Perfused brains were immediately homogenized in Conversion Buffer (PBS 1×, pH 7,4; 0.15 M NaCl; 1% Triton X) with mini-Complete protease inhibitor (Roche) as 10% w/v, and then stored at −80 °C. Fe(III)-TMPyP (1–500 μM) or vehicle control were added to the PMCA substrate just prior to the *in vitro* amplification experiment. Seeds were prepared using brain tissue from Bv109M terminally affected with three different Bv109M-adapted PrP^Sc^ strains, namely Italian, UK and CH1641, derived from the Italian sheep PrP^Sc^ isolate SS7, the UK sheep PrP^Sc^ isolate SCR1^25^, and the experimental PrP^Sc^ isolate CH1641^26^. Tissues were homogenized in PBS (10% w/v), diluted 1:10 and 1:100 in PMCA substrate and either frozen at −20 °C or immediately subjected to PMCA. Samples were processed for 32 continuous cycles of PMCA, consisting of 20 s pulse sonication at 80% power output using a Misonix S3000 sonicator, followed by incubation for 30 min at 37 °C. All samples were analyzed by PK digestion and Western Blotting as previously reported^60^. Blots were probed with anti-PrP monoclonal antibody SAF84 (a.a. 167–173 Bank vole PrP sequence; 1.2 μg/mL), followed by horseradish peroxidase-conjugated anti-mouse immunoglobulin (Pierce Biotechnology, Rockford, IL). Proteinase K (PK)-resistant PrP bands were visualized using the chemiluminescence method (SuperSignal Femto, Pierce), detected by the VersaDoc imaging system (Bio-Rad) and quantified by QuantityOne software (Bio-Rad). The amplification factor was calculated by quantifying the amount of PrP^Sc^ in post-PMCA 1:100 diluted samples (Y) and in the 1:10 frozen dilution (X), taking into account the different dilution factor within the two, using the formula (Y/X) × 10. The amplification factor was then calculated as the mean value (±standard deviation) of independent samples.

### Prion-infected neuroblastoma cells

N2a cells (a subclone of N2a cells infected with the 22L prion strain) were propagated for 5–7 passages to stabilize PrP^Sc^ levels. Cells were grown in culturing medium [Dulbecco’s Minimal Essential Media (DMEM), 10% heat-inactivated fetal bovine serum (Δ56-FBS), Non-Essential Amino Acids (NEAA) and Penicillin/Streptomycin (Pen/Strep)]. To test anti-prion activity of Fe(III)-TMPyP, cells were plated on day 1 in 24-well plates at approximately 60% confluence, in presence or absence of relevant compound (usually at concentrations between 0.1 and 10 μM). On days 2 and 4, 100% medium was changed, adding fresh compound. On day 3, each well was split 1:2 in medium containing fresh compound. In order to avoid the use of trypsin, cells were detached by adding medium directly onto the surface of the well, and mechanically dissociated by pipetting gently for 10–15 times. On day 5, cells were collected by adding 100 μl (per well) of lysis buffer (PBS, pH 7.4, 0.5% NP-40, 0.5% TX-100, plus complete EDTA-free Protease Inhibitor Cocktail Tablets, Roche) directly into the well. Proteinase-K (PK) digestion was performed by incubating 100 μl of lysate in a shaking (450 rpm) thermomixer for 1 h at 37 °C with 10 μg/ml of PK. The reaction was stopped by adding 2 mM PMSF, followed by methanol (MeOH) precipitation (10:1 volume ratio of MeOH to sample; each sample incubated for 4 h at −80 °C and then centrifuged at 14.000 rpm × 20 min at 4 °C). The resulting pellet (dried at room temperature to remove any residual MeOH) was boiled in Laemli sample buffer, and subjected to Western blotting. Blots were probed with 6D11 antibody (1:4000) followed by goat anti-mouse IgG (Pierce), and signals were revealed using enhanced chemiluminescence (Luminata, BioRad) and visualized by a Biorad XRS image scanner.

### Patch clamping of HEK293 cells

The spontaneous ion channel activity induced by the ΔCR PrP mutant in HEK293 cells was detected by whole-cell patch clamping as previously described^61^,^62^. Borosilicate electrodes with an electrical resistance of 3–6 MΩ were used. Cells were visualized with the 40X objectives using an Olympus BX51WI microscope equipped with reflected fluorescence, as well as differential interference contrast observation systems. Experiments were conducted at room temperature with the following solutions: Internal: 140 mM Cs-glucuronate, 5 mM CsCl, 4 mM MgATP, 1 mM Na2GTP, 10 mM EGTA, and 10 mM HEPES (pH 7.4 with CsOH); External: 150 mM NaCl, 4 mM KCl, 2 mM CaCl2, 2 mM MgCl2, 10 mM glucose, and 10 mM HEPES (pH 7.4 with NaOH). Data were acquired using a Multiclamp 700B amplifier and pClamp 10 software (Molecular Devices, Foster City, CA), and sampled at 5 kHz with a Digidata 1440 (Molecular Devices). Data were analyzed off-line using the Clampfit 10 software (Molecular Devices).

### Drug-Based Cell Assay (DBCA)

The DBCA was performed as described previously^29^, with minor modifications. Briefly, HEK293 cells expressing ΔCR PrP were cultured at ~60% confluence in 24-well plates on day 1. On day 2, cells were treated with 500 μg/mL of Zeocin and/or Fe(III)-TMPyP (0.1–10 μM) for 72 hr. Medium (containing fresh Zeocin and/or Fe(III)-TMPyP) was replaced every 24 hr. On day 5, cell medium was removed and cells were incubated with 1 mg/mL of 3-(4,5-dimethylthiazol-2-yl)-2,5-diphenyltetrazolium bromide (MTT, Sigma Aldrich, St. Louis, MO) in PBS for 30 min at 37 °C. MTT was carefully removed, and cells were re-suspended in 500 μL of DMSO. Values for each well were obtained by measuring at 570 nm, using a plate spectrophotometer (Biotek).

### Preparation of Aβ oligomers

Synthetic Aβ (1–42) peptide (Karebay Biochem., Rochester, NY) was dissolved in hexafluoro-2-propanol, incubated for 10 min in a bath sonicator at maximum power, centrifuged at 15.000 × g for 1 min, aliquoted, dried, and stored at −80 °C. Before use, the dried film was dissolved using DMSO and diluted to 100 μM in F12 Medium (Invitrogen, Waltham, MA). Oligomers were obtained by incubating the peptide for 16 h at 25 °C. This preparation routinely produces oligomers that elute near the void volume of a Superdex 75 10/300 size exclusion column (GE Healthcare, Little Chalfont, UK), and that react with oligomer-specific antibody A11^33^. Final Aβ oligomer concentrations were considered as monomer equivalents, since the size of the oligomers is heterogeneous.

### Cultured hippocampal neurons

Primary neuronal cultures were derived from the hippocampi of 2-day-old postnatal mice, and cultured as described previously^63^. Neurons were plated on 35-mm dishes (500,000 cells/dish) pre-coated with 25 μg/mL poly-D-lysine (Sigma P6407) in B27/Neurobasal-A medium supplemented with 0.5 mM glutamine, 100 units/mL penicillin, and 100 μg/mL streptomycin (all from Invitrogen). Experiments were performed 12 days after plating. Neurons were pre-treated for 20 min with Fe(III)-TMPyP (10 μM) and then exposed for 20 mins or 3 hr to Aβ oligomers (3 μM). Triton-insoluble fractions (TIF) were analyzed by immunoblot with antibodies against phospho-SFK (Tyr 416) or Fyn. The phospho-SFK antibody detects pY416 in several SFKs, but previous studies showed that PrP^C^-dependent activation of kinases is specific for Fyn. Actin was used as loading control. Subcellular fractionation was performed as reported previously, with minor modifications. Neurons were homogenized using a Potter-Elvehjem homogenizer in 0.32 M ice-cold sucrose buffer (pH 7.4) containing 1 mM HEPES, 1 mM MgCl2, 10 mM NAF, 1 mM NaHCO3, and 0.1 mM PMSF in the presence of protease inhibitors (Complete mini, Roche Applied Science, Penzberg, Germany) and phosphatase inhibitors (PhosSTOP, Roche Applied Science). Samples were centrifuged at 13.000 × g for 15 min to obtain a crude membrane fraction. The pellet was re-suspended in buffer containing 150 mM KCl and 0.5% Triton X-100 and centrifuged at 100,000 × g for 1 hr. The final pellet, referred to as the Triton-insoluble fraction, was re-homogenized in 20 mM HEPES supplemented with protease and phosphatase inhibitors and then stored at −80 °C or directly used in further experiments. Protein concentration in each sample was quantified using the Bradford assay (Bio-Rad), and proteins (5 μg) were then analyzed by Western blotting. Primary antibodies were as follow: anti-GluN2A and anti-GluN2B (both 1:2000; Invitrogen), anti-GluA1 and anti-GluA2 (both 1:1000; Millipore, Billerica, MA), anti-PSD-95 (post-synaptic density protein 95; 1:2000; Cayman Chemical, Ann Arbor, MI), and anti-actin (1:5000; Millipore). Western blots were analyzed by densitometry using Quantity One software (Bio-Rad). All experiments were repeated on at least 4 independent culture preparations (n = 4).

## Additional Information

**How to cite this article**: Massignan, T. *et al*. A cationic tetrapyrrole inhibits toxic activities of the cellular prion protein. *Sci. Rep.* **6**, 23180; doi: 10.1038/srep23180 (2016).

## References

[b1] Colby D. W. & Prusiner S. B. Prions. Cold Spring Harb Perspect Biol 3, a006833, doi: 10.1101/cshperspect.a006833 (2011). 21421910 PMC3003464

[b2] Collinge J. & Clarke A. R. A general model of prion strains and their pathogenicity. Science 318, 930–936 (2007). 17991853 10.1126/science.1138718

[b3] Kawasaki Y. . Orally administered amyloidophilic compound is effective in prolonging the incubation periods of animals cerebrally infected with prion diseases in a prion strain-dependent manner. J Virol 81, 12889–12898, doi: JVI.01563-07 (2007). 17881452 10.1128/JVI.01563-07 PMC2169081

[b4] Wagner J. . Anle138b: a novel oligomer modulator for disease-modifying therapy of neurodegenerative diseases such as prion and Parkinson’s disease. Acta Neuropathol 125, 795–813, doi: 10.1007/s00401-013-1114-9 (2013). 23604588 PMC3661926

[b5] Ghaemmaghami S., Russo M. & Renslo A. R. Successes and challenges in phenotype-based lead discovery for prion diseases. J Med Chem. 57, 6919–6929, doi: 10.1021/jm5001425 (2014). 24762293 PMC4148153

[b6] Li J., Browning S., Mahal S. P., Oelschlegel A. M. & Weissmann C. Darwinian evolution of prions in cell culture. Science 327, 869–872, doi: science.1183218 (2010). 20044542 10.1126/science.1183218 PMC2848070

[b7] Oelschlegel A. M. & Weissmann C. Acquisition of drug resistance and dependence by prions. PLoS Pathog 9, e1003158, doi: 10.1371/journal.ppat.1003158PPATHOGENS-D-12-02364 (2013). 23408888 PMC3567182

[b8] Mallucci G. . Depleting neuronal PrP in prion infection prevents disease and reverses spongiosis. Science 302, 871–874 (2003). 14593181 10.1126/science.1090187

[b9] Brandner S. . Normal host prion protein (PrPC) is required for scrapie spread within the central nervous system. Proc Natl Acad Sci USA 93, 13148–13151 (1996). 8917559 10.1073/pnas.93.23.13148 PMC24061

[b10] Biasini E., Turnbaugh J. A., Unterberger U. & Harris D. A. Prion protein at the crossroads of physiology and disease. Trends Neurosci 35, 92–103, doi: S0166-2236(11)00173-1 (2012). 22137337 10.1016/j.tins.2011.10.002 PMC3273588

[b11] Lauren J., Gimbel D. A., Nygaard H. B., Gilbert J. W. & Strittmatter S. M. Cellular prion protein mediates impairment of synaptic plasticity by amyloid-beta oligomers. Nature 457, 1128–1132 (2009). 19242475 10.1038/nature07761 PMC2748841

[b12] Resenberger U. K. . The cellular prion protein mediates neurotoxic signalling of beta-sheet-rich conformers independent of prion replication. EMBO J 30, 2057–2070, doi: emboj201186 (2011). 21441896 10.1038/emboj.2011.86 PMC3098494

[b13] Biasini E. & Harris D. A. Targeting the cellular prion protein to treat neurodegeneration. Future Med Chem. 4, 1655–1658, doi: 10.4155/fmc.12.114 (2012). 22924502

[b14] Iraci N., Stincardini C., Barreca M. L. & Biasini E. Decoding the function of the N-terminal tail of the cellular prion protein to inspire novel therapeutic avenues for neurodegenerative diseases. Virus Res. 207, 62–68, doi: 10.1016/j.virusres.2014.10.015 (2015). 25456402

[b15] Sim V. L. Prion disease: chemotherapeutic strategies. Infect Disord Drug Targets 12, 144–160, doi: IDDT-EPUP-20120314-001 (2012). 22420513 10.2174/187152612800100161

[b16] Caughey W. S., Raymond L. D., Horiuchi M. & Caughey B. Inhibition of protease-resistant prion protein formation by porphyrins and phthalocyanines. Proc Natl Acad Sci USA 95, 12117–12122 (1998). 9770449 10.1073/pnas.95.21.12117 PMC22794

[b17] Nicoll A. J. . Pharmacological chaperone for the structured domain of human prion protein. Proc Natl Acad Sci USA 107, 17610–17615, doi: 1009062107 (2010). 20876144 10.1073/pnas.1009062107 PMC2955083

[b18] Xiao C. Q. . Comprehensive study of the interaction between a potential antiprion cationic porphyrin and human prion protein at different pH by using multiple spectroscopic methods. J Pharm Sci. 102, 1076–1085, doi: 10.1002/jps.23420 (2013). 23280556

[b19] Priola S. A., Raines A. & Caughey W. S. Porphyrin and phthalocyanine antiscrapie compounds. Science 287, 1503–1506, doi: 8296 (2000). 10688802 10.1126/science.287.5457.1503

[b20] Kocisko D. A. . A porphyrin increases survival time of mice after intracerebral prion infection. Antimicrob Agents Chemother 50, 759–761, doi: 50/2/759 (2006). 16436739 10.1128/AAC.50.2.759-761.2006 PMC1366918

[b21] Kuwata K. . Hot spots in prion protein for pathogenic conversion. Proc Natl Acad Sci USA 104, 11921–11926, doi: 0702671104 (2007). 17616582 10.1073/pnas.0702671104 PMC1924567

[b22] Codd E. E., Mabus J. R., Murray B. S., Zhang S. P. & Flores C. M. Dynamic mass redistribution as a means to measure and differentiate signaling via opioid and cannabinoid receptors. Assay Drug Dev Technol. 9, 362–372, doi: 10.1089/adt.2010.0347 (2011). 21323580

[b23] Tran E., Sun H. & Fang Y. Dynamic mass redistribution assays decode surface influence on signaling of endogenous purinergic P2Y receptors. Assay Drug Dev Technol. 10, 37–45, doi: 10.1089/adt.2011.0392 (2012). 22066912 PMC3277731

[b24] Saa P., Castilla J. & Soto C. Cyclic amplification of protein misfolding and aggregation. Methods Mol Biol 299, 53–65, doi: 1-59259-874-9:053 (2005). 15980595 10.1385/1-59259-874-9:053

[b25] Di Bari M. A. . The bank vole (Myodes glareolus) as a sensitive bioassay for sheep scrapie. J Gen Virol. 89, 2975–2985, doi: 10.1099/vir.0.2008/005520-0 (2008). 19008382

[b26] Konold T. . Further characterisation of transmissible spongiform encephalopathy phenotypes after inoculation of cattle with two temporally separated sources of sheep scrapie from Great Britain. BMC Res Notes. 8, 312, doi: 10.1186/s13104-015-1260-3 (2015). 26205536 PMC4618938

[b27] Li A. . Neonatal lethality in transgenic mice expressing prion protein with a deletion of residues 105–125. Embo J 26, 548–558 (2007). 17245437 10.1038/sj.emboj.7601507 PMC1783448

[b28] Solomon I. H. . An N-terminal polybasic domain and cell surface localization are required for mutant prion protein toxicity. J Biol Chem 286, 14724–14736, doi: M110.214973 (2011). 21385869 10.1074/jbc.M110.214973 PMC3077669

[b29] Massignan T. . A novel, drug-based, cellular assay for the activity of neurotoxic mutants of the prion protein. J Biol Chem 285, 7752–7765, doi: M109.064949 (2010). 19940127 10.1074/jbc.M109.064949 PMC2844219

[b30] Massignan T., Biasini E. & Harris D. A. A Drug-Based Cellular Assay (DBCA) for studying cytotoxic and cytoprotective activities of the prion protein: A practical guide. Methods 53, 214–219, doi: 10.1016/j.ymeth.2010.11.005 (2011). 21115124 PMC3384733

[b31] Um J. W. & Strittmatter S. M. Amyloid-beta induced signaling by cellular prion protein and Fyn kinase in Alzheimer disease. Prion 7, 37–41, doi: 22212 (2013). 22987042 10.4161/pri.22212 PMC3609048

[b32] Um J. W. . Alzheimer amyloid-beta oligomer bound to postsynaptic prion protein activates Fyn to impair neurons. Nat Neurosci 15, 1227–1235, doi: nn.3178 (2012). 22820466 10.1038/nn.3178 PMC3431439

[b33] Fluharty B. R. . An N-terminal fragment of the prion protein binds to amyloid-beta oligomers and inhibits their neurotoxicity *in vivo*. J Biol Chem 288, 7857–7866, doi: M112.423954 (2013). 23362282 10.1074/jbc.M112.423954 PMC3597823

[b34] Berry D. B. . Drug resistance confounding prion therapeutics. Proc Natl Acad Sci USA 110, E4160–4169, doi: 10.1073/pnas.1317164110 (2013). 24128760 PMC3816483

[b35] Giles K. . Different 2-Aminothiazole Therapeutics Produce Distinct Patterns of Scrapie Prion Neuropathology in Mouse Brains. J Pharmacol Exp Ther. 355, 2–12, doi: 10.1124/jpet.115.224659 (2015). 26224882 PMC4576665

[b36] Lasmezas C. & Zhou M. Newly defined toxic alpha-helical prion protein monomer: implications for other neurodegenerative diseases? Expert Rev Proteomics. 9, 233–235, doi: 10.1586/epr.12.26 (2012). 22809201

[b37] Zhou M., Ottenberg G., Sferrazza G. F. & Lasmezas C. I. Highly neurotoxic monomeric alpha-helical prion protein. Proc Natl Acad Sci USA 109, 3113–3118, doi: 10.1073/pnas.1118090109 (2012). 22323583 PMC3286986

[b38] Trevitt C. R. & Collinge J. A systematic review of prion therapeutics in experimental models. Brain 129, 2241–2265, doi: awl150 (2006). 16816391 10.1093/brain/awl150

[b39] Maurer M. S. . Tafamidis in transthyretin amyloid cardiomyopathy: effects on transthyretin stabilization and clinical outcomes. Circ Heart Fail. 8, 519–526, doi: 10.1161/CIRCHEARTFAILURE.113.000890 (2015). 25872787

[b40] Scott L. J. Tafamidis: a review of its use in familial amyloid polyneuropathy. Drugs 74, 1371–1378, doi: 10.1007/s40265-014-0260-2 (2014). 25022953

[b41] Poncet-Montange G. . A survey of antiprion compounds reveals the prevalence of non-PrP molecular targets. J Biol Chem 286, 27718–27728, doi: M111.234393 (2011). 21610081 10.1074/jbc.M111.234393 PMC3149362

[b42] Kamatari Y. O., Hayano Y., Yamaguchi K., Hosokawa-Muto J. & Kuwata K. Characterizing antiprion compounds based on their binding properties to prion proteins: implications as medical chaperones. Protein Sci. 22, 22–34, doi: 10.1002/pro.2180 (2013). 23081827 PMC3575857

[b43] Baral P. K. . Structural basis of prion inhibition by phenothiazine compounds. Structure 22, 291–303, doi: 10.1016/j.str.2013.11.009 (2014). 24373770

[b44] Yamasaki T., Suzuki A., Hasebe R. & Horiuchi M. Comparison of the anti-prion mechanism of four different anti-prion compounds, anti-PrP monoclonal antibody 44B1, pentosan polysulfate, chlorpromazine, and U18666A, in prion-infected mouse neuroblastoma cells. PLoS One 9, e106516, doi: 10.1371/journal.pone.0106516 (2014). 25181483 PMC4152300

[b45] Kocisko D. A. . New inhibitors of scrapie-associated prion protein formation in a library of 2000 drugs and natural products. J Virol 77, 10288–10294 (2003). 12970413 10.1128/JVI.77.19.10288-10294.2003 PMC228499

[b46] Linden R. . Physiology of the prion protein. Physiol Rev 88, 673–728, doi: 88/2/673 (2008). 18391177 10.1152/physrev.00007.2007

[b47] Solomon I. H., Schepker J. A. & Harris D. A. Prion neurotoxicity: insights from prion protein mutants. Curr Issues Mol Biol 12, 51–62 (2009). 19767650 PMC4821541

[b48] Baumann F. . Lethal recessive myelin toxicity of prion protein lacking its central domain. Embo J 26, 538–547 (2007). 17245436 10.1038/sj.emboj.7601510 PMC1783444

[b49] Shmerling D. . Expression of amino-terminally truncated PrP in the mouse leading to ataxia and specific cerebellar lesions. Cell 93, 203–214. (1998). 9568713 10.1016/s0092-8674(00)81572-x

[b50] Christensen H. M. & Harris D. A. A deleted prion protein that is neurotoxic *in vivo* is localized normally in cultured cells. J Neurochem 108, 44–56 (2009). 19046329 10.1111/j.1471-4159.2008.05719.x

[b51] Biasini E. . A mutant prion protein sensitizes neurons to glutamate-induced excitotoxicity. J Neurosci 33, 2408–2418, doi: 33/6/2408 (2013). 23392670 10.1523/JNEUROSCI.3406-12.2013 PMC3711660

[b52] Chen S., Yadav S. P. & Surewicz W. K. Interaction between human prion protein and amyloid-beta (Abeta) oligomers: role OF N-terminal residues. J Biol Chem 285, 26377–26383, doi: 10.1074/jbc.M110.145516 (2010). 20576610 PMC2924066

[b53] Sonati T. . The toxicity of antiprion antibodies is mediated by the flexible tail of the prion protein. Nature 501, 102–106, doi: nature12402 (2013). 23903654 10.1038/nature12402

[b54] Martinez J. . PrP charge structure encodes interdomain interactions. Sci Rep. 5, 13623, doi: 10.1038/srep13623 (2015). 26323476 PMC4555102

[b55] Freir D. B. . Interaction between prion protein and toxic amyloid beta assemblies can be therapeutically targeted at multiple sites. Nat Commun. 2, 336, doi: 10.1038/ncomms1341 (2011). 21654636 PMC3156817

[b56] Spevacek A. R. . Zinc drives a tertiary fold in the prion protein with familial disease mutation sites at the interface. Structure, doi: S0969-2126(12)00457-1 (2012). 10.1016/j.str.2012.12.002 PMC3570608 23290724

[b57] Negro A. . Susceptibility of the prion protein to enzymic phosphorylation. Biochem Biophys Res Commun. 271, 337–341 (2000). 10799298 10.1006/bbrc.2000.2628

[b58] Castilla J., Saa P., Hetz C. & Soto C. *In vitro* generation of infectious scrapie prions. Cell 121, 195–206, doi: S0092-8674(05)00156-X (2005). 15851027 10.1016/j.cell.2005.02.011

[b59] Cosseddu G. M. . Ultra-efficient PrP(Sc) amplification highlights potentialities and pitfalls of PMCA technology. PLoS Pathog 7, e1002370, doi: 10.1371/journal.ppat.1002370 (2011). 22114554 PMC3219717

[b60] Vanni I. . *In vitro* replication highlights the mutability of prions. Prion 8, 154–160, doi: 10.4161/pri.28468 (2014). 24618479 PMC7030905

[b61] Solomon I. H., Biasini E. & Harris D. A. Ion channels induced by the prion protein: mediators of neurotoxicity. Prion 6, 40–45, doi: 18627 (2012). 22453177 10.4161/pri.6.1.18627 PMC3338964

[b62] Solomon I. H., Huettner J. E. & Harris D. A. Neurotoxic mutants of the prion protein induce spontaneous ionic currents in cultured cells. J Biol Chem 285, 26719–26726, doi: M110.134619 (2010). 20573963 10.1074/jbc.M110.134619 PMC2924115

[b63] Balducci C. . Synthetic amyloid-beta oligomers impair long-term memory independently of cellular prion protein. Proc Natl Acad Sci USA 107, 2295–2300 (2010). 20133875 10.1073/pnas.0911829107 PMC2836680

